# Incorporating Hypnosis into Pediatric Clinical Encounters

**DOI:** 10.3390/children4030018

**Published:** 2017-03-16

**Authors:** Robert A. Pendergrast

**Affiliations:** Department of Pediatrics, Medical College of Georgia, Augusta University, 1120 15th Street, Augusta, GA 30912, USA; rpenderg@augusta.edu; Tel.: +1-706-721-2457; Fax: +1-706-721-3912

**Keywords:** hypnosis, pediatric hypnosis, pediatric skills development, self-hypnosis, primary care, therapeutic use of language, hypnosis consult, practice style

## Abstract

Increasing numbers of licensed health professionals who care for children have been trained in clinical hypnosis. The evidence base for the safety and efficacy of this therapeutic approach in a wide variety of conditions is also growing. Pediatricians and other health professionals who have received training may wish to apply these skills in appropriate clinical scenarios but still may be unsure of the practical matters of how to incorporate this skill-set into day to day practice. Moreover, the practical application of such skills will take very different forms depending on the practice setting, types of acute or chronic conditions, patient and family preferences, and the developmental stages of the child or teen. This article reviews the application of pediatric clinical hypnosis skills by describing the use of hypnotic language outside of formal trance induction, by describing natural trance states that occur in children and teens in healthcare settings, and by describing the process of planning a clinical hypnosis encounter. It is assumed that this article does not constitute training in hypnosis or qualify its readers for the application of such skills; rather, it may serve as a practical guide for those professionals who have been so trained, and may serve to inform other professionals what to expect when referring a patient for hypnotherapy. The reader is referred to specific training opportunities and organizations.

## 1. Introduction

Though called by different names, there has been interest in the concept of clinical hypnosis by health professionals caring for children at least as far into Western history as the 18th century. This has been reviewed briefly by Kohen and Kaiser [1] and in more detail in Kohen and Olness’ Hypnosis and Hypnotherapy in Children [2]. Since the 1960s, there has been a rapid expansion of dissemination of knowledge in the field, numbers of professionals receiving training and certification, and the development of a specifically pediatric curriculum. Since 1987, annual pediatric training workshops have been offered, initially under the auspices of the Society for Developmental and Behavioral Pediatrics, and since 2010 by the National Pediatric Hypnosis Training Institute, both American organizations. In Germany, the Kindertagung (the largest child hypnosis congress in the world) has been held periodically since 1990, with thousands of participants and an international faculty. There are excellent textbooks in the field, including Hypnosis and Hypnotherapy in Children [2], and Therapeutic Hypnosis with Children and Adolescents [3]. The American Journal of Clinical Hypnosis has published special issues on pediatric specific themes [4]. However, despite increasingly rich training and education resources, the number of licensed health professionals regularly utilizing hypnosis in practice is relatively small compared, say, to other similar skill sets such as active listening, motivational interviewing, or mindfulness training for example. Even after training, a practitioner may face the difficulty of local access to mentoring or modeling of the practical matters of incorporating hypnosis into pediatric clinical encounters. While not a substitute for training, this article will review some of those matters of practical application, the use of language, and may demystify the hypnosis referral process for other professionals.

## 2. Utilizing and Planning

A clinician who wishes to be purposeful in utilizing hypnosis in clinical encounters can begin by simply noticing the number of ways that children demonstrate spontaneous trance, or display behaviors that may be interpreted or understood as spontaneous self-hypnosis skills. While these are not quite conscious or intentional, they are nonetheless common in the context of everyday clinical encounters [5]. These quite natural states may take the form of imaginative language or play during a clinical encounter, intense focus or concentration on an activity, game, book, or puzzle, intense (and even fearful) focus on a physical sensation or injury, or distracted daydreaming that serves the purpose of adaptive dissociation for the child in an otherwise unpleasant situation. The fact that natural trance states occur so frequently in children in healthcare settings leads to the inescapable conclusion that *all* clinicians, whether trained in hypnosis or not, must be very careful with word choices around children. During any trance state, a person is much more likely to accept a suggestion as true (and act upon it); even unintentional suggestions or developmentally misinterpreted statements may have a larger than expected impact on a child [6]. For example, a clinician attempting humor with a 5-year-old with a wrist fracture may say something like “it’s OK, you won’t miss using that arm for a while….” While an adult would be able to process such language as jest, a child in the trance of pain and injury may hear those words as suggestions for disuse or loss of the arm. Both children and adults interpret suggestions made during a trance state very literally and concretely. The concept of “trance logic” implies that during trance, a person may have very limited reality testing and would likely accept and act upon whatever suggestion is offered [7]. A natural trance state may be generated by a variety of healthcare encounters, such as an illness visit, awaiting an immunization, pre-operative preparation, or hearing an interpretation of lab tests or radiology results. Clinicians in these settings must think carefully about their choice of words and the effect of unintentional suggestions.

Conversely, natural trance states give clinicians the opportunity to decide when and how to utilize the child’s demonstrated strengths by the purposeful use of language to build positive expectancy and positive outcomes. Thoughtful use of hypnotic language includes choice of words, tone of voice, and reflective listening. The decision to utilize the child’s own natural self-hypnosis may be implemented immediately (e.g. “I’m impressed by how you are helping yourself right now” for a child with Tourette’s who is not having tics in the office), or implemented later when appropriate in the conversation (e.g., “remember a few minutes ago when you were enjoying your new book? Did you know *you can do that* while the nurse is giving you medicine in your arm so it doesn’t have to *bother* you so much?”).

Natural trance states in children can be understood as the child’s spontaneous and subconscious attempt to organize their experiences (especially novel and perhaps frightening experiences) and make new connections in four areas of self-regulation: cognitive, affective/emotional, physiologic, and behavioral. When clinicians are attentive and prepared, they can notice and then facilitate the child’s nascent self-regulatory capacity by “being hypnotic” in a clinical conversation. For example, an observant clinician dealing with a child with a delayed sleep onset disorder may be watching for a child’s attempts at emotional self-regulation and learning about how the child might self-soothe from anxiety symptoms. Based on moment-to-moment observation of the child, the clinical interview might include something like “I notice you took a nice deep breath just now… I wonder why/how your brain chose *right now* to do that?” Or to access the child’s ability for physiologic self-regulation, a clinician could ask “what have you done in the past to help that tummy feel better when stress or worry had made it uncomfortable?” This approach is quite different from one that would take a clinical history, view all of that as devoid of trance or hypnotic phenomena, and then verbally move into a new section of the appointment understood or even labeled as “doing hypnosis.” The concept of utilizing children’s trance as a way of enhancing self-regulatory skills has been expertly reviewed by Kaiser [8].

This approach to enhancing children’s self-regulatory capacity by utilizing hypnotic language without “doing hypnosis” can be characterized as noticing and cultivating the natural hypnotic state, or “using the hypnosis” in the encounter. It usually occurs in the context of taking a clinical history. But the history taking process can and should be more of a conversation than a question and answer checklist, in which clinical questions and open-ended reflection are designed to help the child begin to reframe their experiences, expand their cognitive capacity by attaching new labels to experience, and expand their emotional fluency and self-awareness. The clinician can be extremely helpful to children and teens by simply using reflective listening skills to enhance self-awareness, knowing that self-awareness is a foundational first step to self-regulation. For example, a child may present to the medical office with both chronic abdominal pain and separation anxiety, but has never comprehended the connection between the two. The clinical conversation helps the child to make sense of his or her disconnected experiences and understand that they are not separate phenomena. Because of curiosity and novelty, this conversation is often hypnotic, but is not “doing hypnosis” in the sense that there is a formal sequence of invitation, induction or any script. It is important in this type of clinical conversation for the clinician to provide observations of the co-incidence of phenomena in the child’s life, but in the end each patient must make the connections for themselves by responding to questions like “tell me what you make of that….”

Complementary to the concept of utilizing natural trance states, it is equally important for clinicians to be deliberate in planning or designing hypnotic encounters, especially before entering a consult in which the reason for the appointment is already known. This implies intentional planning, a carefully crafted encounter with a patient that is more likely to have been thought out ahead of time, reviewing a clinical history that is (mostly) known and planning the structure and content of an appointment, including the type of induction and the metaphors/suggestions that would be most appropriate based on the clinical history. It may be helpful for the clinician to outline in writing some expected steps of the appointment, following a predicted sequence: interim history, today’s therapeutic goals, induction technique, intensification strategy, suggestions/metaphors, post-hypnotic suggestions, alerting and ratification, and follow-up plan. It involves giving therapeutic, specific suggestions related to specific goals for that particular clinical encounter. (Induction and intensification techniques and the specific use of hypnotic suggestion and therapeutic metaphor are beyond the scope of this article.) Thoughtful planning of each of the expected steps of the appointment can make a positive outcome more likely. Still, even when carefully planned and conceived ahead of time, in any given appointment a clinician must be prepared to flexibly follow cues from the child, adapting strategies based on how the child demonstrates readiness for help in cultivating these skills.

Whether “doing hypnosis” or “noticing and utilizing and being hypnotic”, clinicians must recognize in this model that patients, especially children, bring trance with them into the setting without being aware of it. Trance is what the brain does naturally when it encounters something for the first time, giving attention to novelty: “I never saw that before,” “I never thought of it that way before,” “I never met anyone quite like you before,” or even “I never had a pain quite like this before.” Trance can be elevating and activating (like a 6-month old reaching for a cardboard picture book), or it can be terrible like the fight or flight response of someone who encounters a phobic object. Within that trance, there is often an openness to change, a readiness to see and experience the world differently, that is, to learn. That may occur with or without the patient having any cultivated self-hypnosis skills, but the attentive clinician will often find that children and teens arrive at appointments with some skills they have already discovered for themselves in this area.

Thus, whether the encounter is spontaneous or planned, clinicians bring their own skill sets and attentiveness to the encounter, ready to cultivate change for the patient, but only when and if the child or teen allows that to happen. In any encounter where the clinician is doing something formally or informally planned to be hypnotic or to evoke hypnosis in the patient, nothing happens at all unless the patient is willing and open to this. In this model, one may see hypnosis as an interpersonal interaction by which the clinician cultivates a trance that the patient makes accessible, for the purpose of a specific health related outcome.

## 3. Primary Care Pediatrics

In the primary care pediatric setting, there are four areas in which a clinician’s choice of words can intersect with a child’s curiosity and become a spontaneously hypnotic encounter that facilitates positive outcomes. They are: developmental mastery, positive expectation, behavior change/motivation, and discovering self-regulation.

*Developmental mastery*: Even during well-child checkups for infants and toddlers, a clinician can reinforce the child’s own growing sense of competence or the maternal-infant dyad’s resilience by making the screening history conversational. For example, pointing out to a new mother how alert and attentive a nursing infant is, talking with parents about how their newborn is constantly learning, commenting on how focused is the 6-month old reaching for a cardboard picture book (demonstrating trance, encountering novelty). In these settings, the mother is often also in trance focusing on her child, so these are opportune moments for positive comments and suggestions regarding the multitude of opportunities that parents and caregivers have to interact with the child in ways that build resilience and developmental mastery.

Pediatric practitioners have daily opportunities to reinforce the child’s growing developmental competence. The verbal content of the well-child history changes with age and developmental stages, but at each age, the words that a clinician chooses can be powerfully reinforcing. The child’s natural trance state in the healthcare encounter creates an opportunity for purposeful suggestions that are specific to the developmental stage of the child or teen. Any pediatric health provider wishing to apply clinical hypnosis must first become fluent in the normal stages of child development and learn how to connect with children in a developmentally appropriate way [9]. For example, a 3-year-old (in Erikson’s stage of autonomy versus shame and doubt) would have his/her developmental mastery reinforced by a comment like, “look how well you climbed onto the exam table *by yourself*! When you were a baby you couldn’t do that!,” or during a checkup saying to a 10-year-old (stage of industry versus inferiority, where competence is the key strength to be developed) “You’re good at lots of things! I wonder how many things you’ll learn to be good at by this time next year?.”

*Positive expectation*: Setting positive expectations for children in pediatric healthcare settings is an important and often neglected challenge. Immunizations, phlebotomy, or the uncertainty and anxiety regarding the physical examination all create strongly negative expectations for children and teens in medical offices. Using distraction and other hypnotic approaches to reduce children’s discomfort in medical offices is a very important subject in its own right and will not be covered in detail in this paper [10]. A growing body of literature supports the idea that measures to reduce pain of immunizations and procedures in children are both important and achievable [11]. Even without structured office interventions to reduce discomfort, each clinician can help children and teens feel more comfortable, physically and emotionally, by careful choice of words. Because children and teens are often in a natural trance state (albeit a negative one full of intense focus on a feared event) in the medical office, a carefully crafted statement that reframes the experience and opens the child to new expectations can be powerful. For example, “I don’t really know just what it will feel like for you, but I do know that you may be surprised at how little it will have to bother you, and how easy it may be for you just to pay attention to something else while the nurse takes good care of that arm over there….” On the other hand, suggestions such as “it’s not going to *hurt*,” can be pro-nociceptive. A child’s thoughts will invariably focus on the word “hurt” and ignore the word “not.” Finding ways to use words to substitute for “pain” and “hurt” is important. Use “bother” in place of “hurt,” and “discomfort” instead of “pain”.

*Behavior change/motivation*: Any primary care office caring for adolescents will present daily opportunities to use hypnotic language to promote behavior change. In this context, there is a large overlap between the use of hypnotic language and the principles of motivational interviewing [12,13]. One of the core concepts of motivational interviewing is Developing Discrepancy. This is the process in which the clinician facilitates the patient’s awareness of his/her current state versus a desired future state. A respectful and empathetic conversation that provokes the teen to think deeply about this discrepancy can often lead to spontaneous trance experiences in the patient. For example, asking a teen, “I wonder what sort of things you picture or think about when you think about yourself one year from now…” can be followed by an expectant silence, in which trance phenomena may be observed. This can be a very useful way to gauge readiness to change. Another key concept of motivational interviewing is that of Exploring Ambivalence. A question such as “will you miss the extra weight when it is gone?” is an implied hypnotic suggestion that the weight will in fact be gone at some future time. Second, it implies that the patient may be ambivalent about it and can (perhaps in spontaneous trance induced by such a novel question) explore the pros and cons of continuing current behaviors versus changing. One can also explore other motivational interviewing domains while the patient is in trance, either from a formal hypnotic induction or spontaneous. A suggestion for future visualization of the self (either with the positive health behavior change or not) can assist the patient in exploring his/her readiness to change, and in affirming his/her own goals. For example, “while these thoughts are on your mind, take a moment to picture yourself, as if on a ‘future TV screen’ and get a sense of what you like about that picture, what you don’t like about that picture, and what choices you can make now that affect that future.” This clinical encounter may be in the mental health office or in the primary care office around issues of health behavior.

*Self-regulation*: Another important way in which purposeful language in outpatient settings can be hypnotic is in facilitating discovery of psychophysiological self-regulation (e.g., breath awareness). Many opportunities for this in clinical care. For example, a brief intervention in the office for a child with anxiety can start with a question like, “Can you put your hand on the place where that worry shows up most in your body?” Most children will readily identify the site of the most troubling somatic symptoms related to stress. A follow-up question can immediately follow: “Would it be OK to learn how to help yourself feel better there?” Teaching the child or teen simple techniques of abdominal breathing and then noticing how they feel differently can be a powerful first step in enhanced self-regulation. It is also important during the clinical encounter to encourage the child or teen to reflect on how they may have already discovered their own methods of physiologic self-regulation. It is not uncommon for a patient to disclose their own techniques to the clinician, such as “I already use my imagination and breathing when I am nervous at bedtime.” The clinician’s task then is to reinforce and ratify the child’s own strengths with statements like “helping you learn to help yourself is going to be easier than I thought, since you are *already good at changing things*”.

## 4. Hospital and Acute Care Settings

Hypnotic language can often be utilized in hospital settings or in preparation for medical procedures [14]. This can be very informal, without a formal induction, and may occur in the routine course of daily work for a pediatric hospitalist or a Child Life professional. A clinician may also set a longer appointment time for more formal hypnotherapy to prepare for an anticipated procedure. Fundamentally, this involves rehearsing the procedure in trance and creating positive expectations. However, any such intervention must be developmentally appropriate, and expecting a preschooler to sit quietly and visualize is not developmentally on target. Such a child could be encouraged to bring a doll or favorite toy with them, and a playful conversation about how well the doll can experience the upcoming procedure may be sufficient, as long as the child is engaged in the imaginative experience. More formal trance induction in an older child could be followed by imaginal desensitization (the “future TV of me”) using imagery and suggestions that reframe and change the child or teen’s expectations. In urgent care and emergency settings, clinicians have opportunities to help children in crisis. The negative trance that children and teens bring with them into that circumstance in fact makes the use of hypnotic language easier, and is also a reminder of the critical importance of choice of words in acute care settings. Our language in such settings is important, because the children are already in a highly suggestible state. During evaluation of an acute injury, it is possible to embed hypnotic suggestions such as “I can tell it hurts, and you know it may keep on keep on hurting until it feels better, or until it doesn’t need to any more….” The likelihood is that the words that “stick” in the child’s consciousness are the words “it feels better,” which is, after all, the desired outcome. Reframing is possible as well, such as “isn’t it nice that your body knows how to use all that good healthy blood to clean out the cut?”

## 5. The Hypnotherapy Consult

Clinicians who are trained and develop their clinical hypnosis practice will invariably be consulted by other clinicians, usually regarding patients who remain symptomatic despite all the usual diagnostic and therapeutic approaches of more conventional pediatric medicine. For such appointments, it is wise to block a longer extended appointment time, at least 45 min, in order to establish rapport, gather the clinical history, explain hypnosis and self-hypnosis to the child and parent(s), clarify the rationale for using hypnotherapy for the troubling symptoms, reassure the parents (more than the child) that a referral for hypnotherapy does not imply that anyone believes the symptoms are not “real,” and then (perhaps) to begin self-hypnosis coaching for the child or teen. Often there is not sufficient time in a first appointment to give adequate attention to the actual use of hypnotherapy, and a quick return appointment is expected.

There are a number of ways that hypnotic language shows up in these appointments even well before anything that looks like hypnosis, and should start during the process of gathering the medical history. The clinician should be alert to signs of trance in the child, and then reflecting their own trance state back to them in several ways. First, find out what they do well, and what resources they have already discovered for wellness and self-regulation. Short phrases that can be useful include:

Tell me about something you do so well, and is so much fun, that you often or usually forget most everything else…

What was going on the last time you felt really good?

Can we talk about that time when you felt really worried but didn’t (throw-up, have a headache, etc.)?

This process always involves the clinician noticing what the patient is saying, how s/he is saying it, the moments which appear to be moments of trance in which s/he is attempting to make sense of connections and novel ideas, and then offering new interpretations of what s/he has said, as a way of opening possibilities to change based on what s/he already knows.

Words and phrases that can help as therapeutic reflection of child’s own trance, specifically planned to cultivate and nurture the child’s own curiosity include:

*How do YOU think is the best way to learn that?*

*You have lots of skills (*in whatever areas already discussed*), are you ready to learn some new things or new ways to help yourself solve that problem with…?*

*How does your bladder know to keep the gate shut while you are sitting in school during the day?*

*Is there a time when pain can be useful?*

*Do you know about the pain switches?*

*I wonder how soon you will be able to turn off the pain switch when it’s not needed…*

*How did you do that?*

Preparation is critical for these consult appointments. The prepared clinician will review prior medical records, referral documents, and his/her own notes for return appointments. This implies that one should keep very detailed notes of appointments. Write down in the medical record the child’s own words that describe their situation; record the history from the parents’ perspective; record observations of the child’s demeanor during the appointment and how that may have changed over the course of the appointment; record their likes and dislikes, things they do well, who they live with, how they are doing in school, and any special circumstances of the family, such as military deployments for example. When the child returns for the second appointment, the prepared clinician has already reviewed these notes in order to build on that rapport and to remember what worked well and what did not work well.

During the appointment, it is advisable to direct most of the conversation to the child, not to the parent. One can explicitly say to the child at the outset that s/he is important, what s/he thinks is important, what s/he says is important, *and* that the parent will also have a turn to talk, because *s/he has some important things to say too*, but that can wait.

This deliberately child-centric posture is, in all likelihood, completely different from any prior experience this child has had with medical professionals, who tend to talk *about* them *to* their parents, but not talking *with* them and acting respectful of what they have to say. This implies to the child that one is confident in them to articulate and express their own wishes and desires. It builds their trust in the clinician, and it most certainly builds their curiosity in what is about to happen because it breaks previously held expectations about conversations with doctors. It creates the space and opportunity to transform old cognitive, emotional, physiologic, or behavioral patterns of self-regulation. It implies a belief that they *themselves* can be an effective change agent for their own issues. This conversation with the child must always be done in a developmentally appropriate way, remembering to talk with a 7-year-old *very* differently than with a 14-year-old.

After planning and preparation is done, the clinician’s behavior toward the patient is that of leading with permission. Leading implies that one has a skill set to use in order to show the patient in a collaborative way *how* they can get to their goals. The patient came to the appointment with a need and did not know on their own how to meet that need; it is the clinician’s job to lead them there. Equally as important, it is the clinician’s job to recognize that all of this happens only if the patient wants it to happen. That is true not just of the hypnotherapy itself, but also of the appointment in general. A useful introductory comment is that “things happen in hypnosis because you want them to happen, not because I want them to happen.” This “leading with permission” posture and behavior continues even during hypnosis, so that the language of suggestions is not prescriptive (e.g., “you will have the pain down to zero by the time I count down from 10”), but permissive (e.g., “I wonder how soon you will find that old pain completely gone… let me know when you get it down to zero…”).

There are pros and cons of keeping parents in the consult room while working with a child or teen [15]. Many clinicians keep the parent in the room for most if not all of the first appointment, enhancing insights into the parent-child interaction and whether it may be a strength or a liability; it can also help to get two different perspectives on the issues at hand. At subsequent appointments, it may not be advisable to keep a parent in the room during hypnotherapy, as a parent can be distracting and cause the child to “perform” in a way that they believe their parents would expect rather than exercising their own autonomy and mastery. Similar to the child’s performance expectations with parents watching, the clinician may also become overly self-conscious with parents watching. A clinician who is personally attached to creating a certain outcome in the clinical encounter or who is consciously or unconsciously trying to please a parent is not likely to give his or her best energy to creating a therapeutic alliance with the child or teen. This can inadvertently sabotage the very clinical outcome the clinician is anxious to demonstrate to the parent. A clinician who is anxious about what a parent is thinking about him or her is not likely to be clinically effective. If a child is very anxious, one may more strongly consider keeping the parent in the room, hoping to gradually wean the child from that anxious attachment to facilitate their mastery of new skills in self-regulation.

Audio recordings of hypnotherapy sessions can be very helpful as a tool to help the child/teen practice at home, and most families now have a smart phone with a voice recorder app, making recording almost effortless. One may want to introduce the idea of using the recording at home with a comment like “you can find this a helpful way to start practicing, until you don’t need the audio anymore.”

On return appointments, the interim history will reveal if things have gone well or not. If the patient’s experience was positive or neutral, they will expect to have a repeat hypnotherapy session with more guidance for self-hypnosis practice at home. It may be advisable on the second visit to use the same hypnotic induction as at the first appointment in order to help reinforce what may still be a fragile practice of self-hypnosis at home. On future appointments with a patient who is making progress, it can be a good idea to teach them a new type of self-induction for themselves at home, to build skills and the opportunity for new types of self-discovery.

If parents or children report that things did not go well after the first appointment, the clinician must gather all the details about what happened in the interim, and re-assess the child/teen’s interest and willingness to use such techniques to feel better. All that may be required is a different approach, along with continued optimism that a child who is motivated to feel well will discover (with expert guidance) a way to enhance their own self-regulatory ability in order to resolve an issue.

## 6. Resources and Training

It is incumbent on all professionals who care for children in healthcare settings to be aware of the power of words in affecting the mental and physical health of children, for better or for worse. Experienced and thoughtful pediatric clinicians are aware of this even without specific training in clinical hypnosis. Over time, most clinicians become more careful in how they speak during clinical encounters, helping children become more comfortable in healthcare settings. However, many of the concepts described in this paper are quite different from widespread practice patterns. A thoughtful clinician wanting to interact with children and teens in such new ways will soon realize that specific and more concentrated training is needed. The annual training workshops conducted by the National Pediatric Hypnosis Training Institute (NPHTI, a United States registered non-profit 501(c)(3) corporation) are directed by experienced clinician educators and achieve consistently high ratings from attendees. More information is available [16]. The American Society of Clinical Hypnosis (ASCH) conducts annual meetings, national and regional workshops, and is the certifying body for health professionals in the United States who have had clinical hypnosis training. There is a small but consistent presence of pediatric offerings the annual workshops by ASCH [17]. Professionals in Europe can avail themselves of the resources and training events from the European Society of Hypnosis [18]. And while one does not become skilled in clinical hypnosis by reading articles or books, there are seminal readings which should form the foundation of basic knowledge of any pediatric clinician. These include Michael Yapko’s book, Trancework [19], and the more specific pediatric titles Hypnosis and Hypnotherapy in Children [2], and Therapeutic Hypnosis with Children and Adolescents [3]. ASCH publishes pediatric specific articles frequently to keep professionals abreast with progress in the field [4]. With frequent attendance at hypnosis training workshops and meetings, a commitment to reading core books and articles, a thoughtful pediatric clinician can not only facilitate transformation for his or her patients, but also transform his or her own professional life and satisfaction.
